# Use of barbed sutures in robotic bariatric bypass surgery: a single-center case series

**DOI:** 10.1186/s12893-019-0563-z

**Published:** 2019-07-23

**Authors:** Jan Henrik Beckmann, Jan-Niclas Kersebaum, Witigo von Schönfels, Thomas Becker, Clemens Schafmayer, Jan Hendrik Egberts

**Affiliations:** Department of General, Visceral-, Thoracic-, Transplantation-, and Pediatric Surgery, Kurt-Semm Center for Laparoscopic and Robotic Assisted Surgery, University Hospital Schleswig Holstein, Campus Kiel, Arnold Heller Strasse 3, 24105 Kiel, Germany

**Keywords:** Gastric bypass, Obesity, Robotic surgery, Roux-en-Y, Self-fixing barbed sutures

## Abstract

**Background:**

Surgical robots are increasingly being used in bariatric surgery. While several studies describe the safety of using barbed sutures in laparoscopic gastric bypass surgery, no reports are available for robotic bariatric procedures. The aim of our article is to determine whether barbed sutures can be used safely in robotic Roux-en-Y bypass (RYGB) surgery.

**Methods:**

This was a single-center, single-surgeon case series of RYGB procedures using the da Vinci® Xi Surgical System (Intuitive Surgery, Sunnyvale, CA, USA) in combination with the use of barbed sutures (Stratafix, Ethicon, Johnson & Johnson, Cincinnati, OH, USA).

**Results:**

Fifty robotic proximal and distal RYGB surgeries were performed. A linear stapled, side-to-side gastrojejunostomy was carried out, whereby the enterotomy was completed with a running resorbable unidirectional barbed suture, Stratafix 2–0. In one case after robotic proximal RYGB, revision surgery was required due to omentum necrosis. Another patient was readmitted due to gastrointestinal bleeding from anastomosis. No anastomotic insufficiencies, no stenoses, or higher-grade complications according to Clavien-Dindo 4a-5 were found.

**Conclusions:**

We found that the use of self-fixing barbed sutures in robotic RYGB is safe. The self-fixing suture enables the robotic surgeon to perform a simple continuous suture without the need for recurrent retraction. Although we are the first to report this procedure, we had a low number of cases and no control group; thus, further studies with a higher level of evidence are required.

**Electronic supplementary material:**

The online version of this article (10.1186/s12893-019-0563-z) contains supplementary material, which is available to authorized users.

## Background

The Roux-en-Y gastric bypass (RYGB) is still one of the most frequently performed bariatric procedures, and is considered to be the standard therapy for morbid obesity especially in the presence of type II diabetes or gastroesophageal reflux disease [1, 2]. Routinely, the operation is performed laparoscopically, whereby various anastomotic techniques are described. The linear stapler anastomosis is widely used and is superior to the circular stapler anastomosis with regard to stenosis rates, wound infections, and operative time [3]. No difference was found with regard to insufficiency rates. The anastomosis can also be completely hand-sewn, which compared to the circular stapler anastomosis results in lower wound infection rates and lower gastrointestinal bleeding rates, within the same operative time and comparable safety [4].

We have been using the linear stapler anastomoses routinely for years. After applying the anastomosis, the enterotomy must be closed with a suture. Laparoscopic knotting and suturing is one of the most demanding laparoscopic procedures that must be mastered to avoid complications. As standard in laparoscopic procedures, we perform a continuous seromuscular running suture with resorbable braided suture material 2–0 Vicryl (Ethicon, Johnson & Johnson, Cincinnati, OH, USA) for the gastrojejunostomy and 3–0 Vicryl for the jejunojejunostomy. The first assistant is responsible for guiding the suture and maintaining the suture tension, as the suture cannot be tightened easily after completion, especially with braided sutures.

Self-fixing barbed suture material makes the laparoscopic knot obsolete and promises a constant thread tension after a single tightening of the barbed suture material. Studies have proven the safety and efficacy of this special suture material in laparoscopy [5–8]. Gys et al. reported a comparable operative time with a shorter anastomosis time in a randomized controlled study [5, 6], while Vidarsson et al. evaluated a cohort of 25,000 patients and found indications of a shortening of the total operating time, also with comparable safety [7]. Recently, Pennestri et al. also described a significantly shorter median operative time in laparoscopic RYGB using the barbed suture in a retrospective analysis [8].

Increasingly, bariatric interventions are also performed with robot-assisted surgery. Meta-analyses and registry studies show that the technology is safe [9–11]. At longer operative times and higher overall costs, robotic surgery seems to be associated with reduced anastomotic insufficiency and stenosis rates – although large randomized studies are still missing [9, 10].

Since 2017, we have been performing RYGB operations primarily with the help of the da Vinci® Xi Surgical System (Intuitive Surgery, Sunnyvale, CA, USA) [12]. Based on our previous experiences with robotic procedures [12–14], we use unidirectional barbed suture material for the running suture of the enterotomy. While barbed sutures are already successfully used in other surgical areas in combination with a surgical robot, for example in urethra and bronchial anastomoses [13, 15], we are not aware of any report on the use of barbed sutures in robotic gastric bypass surgery. Previous studies on robotic bariatric surgery have mostly used vicryl [16–20]. The aim of our uncontrolled study is to determine whether barbed sutures can be used safely in robotic RYGB surgery.

## Methods

We report the results from a single-center, single-surgeon case series of RYGB procedures that have used the da Vinci Xi Surgical System (Intuitive Surgery, Sunnyvale, CA, USA) in combination with the use of barbed sutures since August 2017 until September 2018. The data were collected prospectively. Written patient consent and an ethics vote are available. The postoperative course after robotic proximal gastric bypass, as well as conversion from sleeve to proximal or distal gastric bypass in case of reflux, planned two-stage procedure, or weight regain, was retrospectively analyzed. Patients were assessed for postoperative complications on a daily basis until discharge, and underwent routine outpatient follow-up at day 30 after surgery. Reoperations within the first 30 days were encompassed as primary endpoints. Secondary endpoints were the incidence of postoperative complications according to the Clavien-Dindo classification [21], both procedure-related and general, as well as operative time, robotic time, and length of hospital stay. Results are presented as mean ± standard deviation. Percentages have been added to categorical values such as the Edmonton obesity staging system (EOSS).

### Surgical technique

**Fig. 1:**
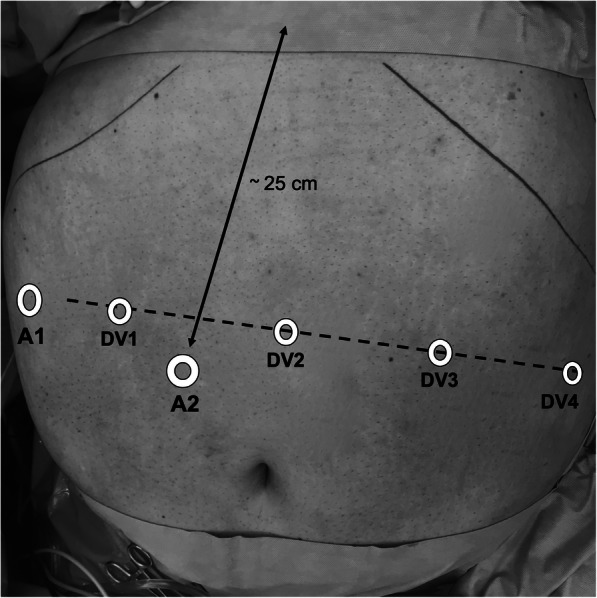
The first access was performed with a 12 mm FIOS First Entry Trocar (Applied Medical) on the right paramedian, about 25 cm below the xiphoid (A2, assist trocar 2). Assist trocar 1 (A1, 12 mm) was placed at the right lateral flank and used for liver retraction. Four 8 mm da Vinci trocars (DV1- DV4) were placed along a line at a distance to each other of about 8 to 10 cm

**Fig. 2:**
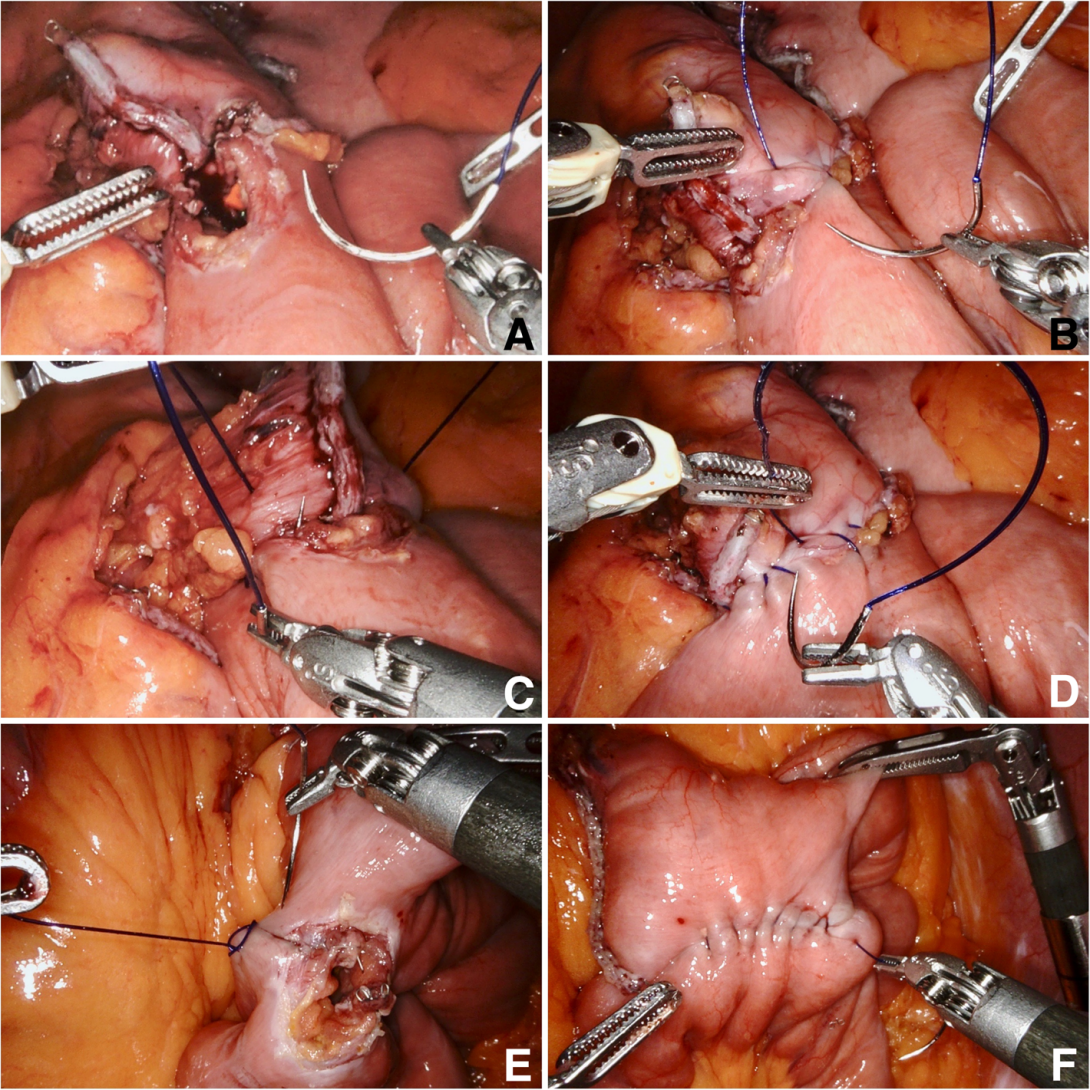
Nodal points of the running suture in robotic Roux-en-Y gastric bypass, **a** Start of gastrojejunostomy with 2–0 Stratafix. Positioning of the small intestine loop with arm 4. **b** Continuous seromuscular suture. **c** Use of a second thread from the right corner. **d** Reversal of the stitch direction for knot-free fixation of the thread. **e** Start of jejunojejunostomy with Stratafix 3–0. **f** Complete closure of the enterotomy before stitch reversal. Exposure by means of the statically used arm 4

The patient was positioned in a 20° anti-Trendelenburg position with the right arm extended, and the left arm at the side. Pneumoperitoneum was established using a 12 mm FIOS First Entry Trocar (Applied Medical, Rancho Santa Margarita, CA, USA) on the right paramedian, about 25 cm below the xiphoid. A 12 mm trocar was positioned at the right subcostal region and used mainly for liver retraction. Four 8 mm da Vinci trocars were positioned as shown in Fig. 1. For instruments, we used a fenestrated bipolar forceps on arm 1, a 30-degree angle optic on arm 2, an Harmonic Ace Curved Shears on arm 3, and a tip-up fenestrated grasper on arm 4 (Intuitive Surgery, Sunnyvale, CA, USA). For suturing, a large needle driver was changed to arm 3. Following careful inspection of the situs, the omentum majus was divided above the colon transversum. After creating a retrogastric tunnel, starting from the lesser curvature approximately 6 cm below the gastroesophageal junction, the stomach pouch was formed using a linear stapler operated by the assistant. The pouch was opened distally by the ultrasonic dissector and the biliopancreatic limb was measured 100 cm from Treitz and opened antimesenterially. The side-to-side gastrojejunostomy was performed with a linear stapler (Echelon Flex, 45 mm, gold cartridge, Ethicon, Johnson & Johnson, Cincinnati, OH, USA) and the enterotomy was closed using a continuous seromuscular suture from each corner, starting with a 15 cm, unidirectional 2–0 Stratafix (Ethicon, Johnson & Johnson, Cincinnati, OH, USA) thread (Fig. 2a-d). Arms 1 and 3 were used dynamically for sewing, while arm 4 was used more statically to optimize the position of the small bowel loop. The first stitch was performed in the left lateral corner of the enterotomy, with inclusion of the staple line (Fig. 2a,b). After completion of the half anastomosis, a second thread was started from the right corner (Fig. 2c). The last 2–3 stitches with each thread were guided in opposite directions to anchor the suture in the tissue and to dispense with the need for a knot (Fig. 2d). The thread should be cut as short as possible to avoid local adhesions. Proximal to the anastomosis, the small intestine was separated with the same linear stapler (Echelon Flex, 45 mm, blue cartridge, Ethicon, Johnson & Johnson, Cincinnati, OH, USA). A Roux limb of 150 cm was measured and a side-to-side linear stapled jejunojejunostomy was created, again using the 45 mm linear stapler (blue cartridge). The enterotomy was closed by a single running suture with Stratafix 3–0 (Fig. 2e,f).

After completing the anastomoses, a methylene blue test of the gastrojejunostomy, the check for blood dryness, the repositioning of the omentum majus, and the installation of a drainage were regularly carried out.

## Results

From August 2017 to September 2018, the surgeon performed 50 RYGB procedures with the da Vinci Xi Surgical System using unidirectional Stratafix sutures. Besides 37 primary proximal RYGB surgeries, four proximal and nine distal RYGB operations were performed as ReDo procedures.

The number of procedures performed, gender, age at surgery, preoperative weight, BMI, and Edmonton obesity staging system (EOSS) are shown in Table 1. Results such as the duration of surgery, console time, length of hospital stay, percentage weight loss, number of revision surgeries, and complications within the first 30 days, according to the Clavien-Dindo classification [21], are listed in the Additional file 1. The complete dataset is listed in the Additional file 2.

**Table 1:** Number of procedures performed, gender, age at surgery, preoperative weight, BMI, and classification of comorbidities according to the Edmonton obesity staging system (EOSS)

	n	Female	Male	Age, y	Weight, kg	BMI, kg/m^2^	EOSS 1	EOSS 2	EOSS 3	EOSS 4
Robotic prox. RYGB	37	28 (76%)	9 (24%)	43.8 ± 12.3	139.3 ± 18.0	47.0 ± 4.2	3 (8%)	16 (43%)	14 (38%)	4 (11%)
Robotic prox. RYGB ReDo	4	3 (75%)	1 (25%)	51.6 ± 10.5	106.8 ± 21.7	36.6 ± 9.4	0	2 (50%)	2 (50%)	0
Robotic dist. RYGB ReDo	9	5 (56%)	4 (44%)	45.2 ± 11.8	142.6 ± 26.4	48.0 ± 6.1	1 (11%)	3 (33%)	5 (56%)	0
All	50	36 (72%)	14 (28%)	44.7 ± 12.1	137.3 ± 21.6	46.4 ± 5.7	4 (8%)	21 (42%)	21 (42%)	4 (8%)

Values are mean ± standard deviation. Percentages have been added to categorical values. Robotic prox. RYGB: da Vinci proximal Roux-en-Y gastric bypass; Robotic prox. RYGB ReDo: da Vinci proximal Roux-en-Y gastric bypass as secondary procedure; Robotic dist. RYGB ReDo: da Vinci distal Roux-en-Y gastric bypass as secondary procedure

No complications occurred intraoperatively. The running sutures for closing the enterotomy were performed without any problems in all cases. In particular, there were no ruptures of the thread.

**Fig. 3:**
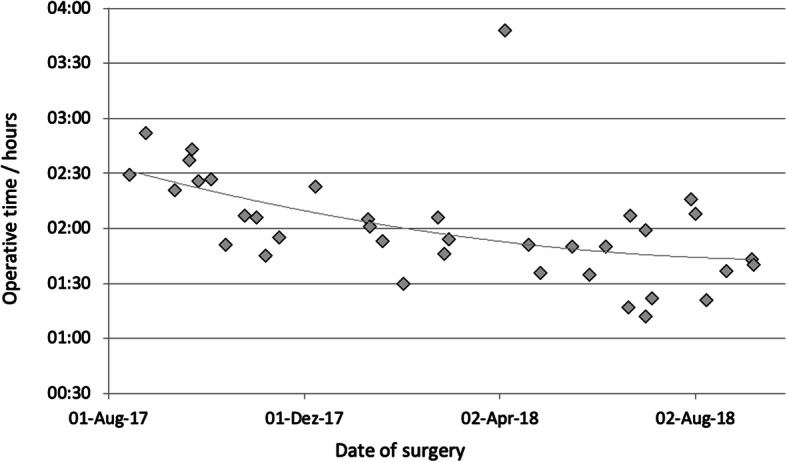
Operative times in hours of primary proximal robotic Roux-en-Y gastric bypass procedures listed by date of surgery. Implemented trendline

The average operation time was 127 min. Twenty-nine minutes were needed to insert the trocars, place the liver retractor, local adhesiolysis, dock the surgical robot, and finally close the abdominal wall, including skin sutures. In one case, with chronic fistula of the proximal staple line 14 months after sleeve gastrectomy, the indication for reconstruction into a proximal bypass was given. The operation and further course were without any problems. This case explains the longer operation times with high standard deviation in the robotic proximal RYGB ReDo group (*n* = 4). The detailed operation times are shown in Fig. 3.

Thirty days postoperatively, a mean ± standard deviation excess weight loss of 21.9 ± 7.5% was recorded after proximal gastric bypass. Ideal body weight was calculated as that equivalent to a BMI of 25 kg/m^2^. The follow-up rate was 98%.

In one case with abnormal infection parameters, a revision operation was performed after proximal RYGB. The cause was a partial omentum necrosis. After partial omentectomy, the patient was discharged on the eighth postoperative day without further complications. Another patient was readmitted 12 days after proximal RYGB with gastrointestinal bleeding. Gastroscopy showed an ulcer in the anastomosis area without active bleeding (Forrest III). No further bleeding occurred under conservative therapy. Blood transfusion was not necessary. The patient was discharged after five days and no more complaints were reported. There were no additional higher-grade complications, in particular no anastomotic insufficiencies and no stenosis.

## Discussion

The role of the surgical robot in obesity surgery is still controversially discussed. At higher costs and overall longer operation times, meta-analyses indicate reduced complication rates, but larger and randomized controlled studies are still missing [9–12]. In our clinic, the robot has been used in gastric bypass operations since the beginning of 2017, using a technique largely comparable to laparoscopy. After linear side-by-side anastomosis, the remaining opening is closed by a continuous seromuscular suture. While in the conventional laparoscopic technique the first assistant guides the suture after each stitch and keeps it taut, these steps cannot be carried out in the same sequence using robots because the fourth arm is used mainly for static activities. In principle, the thread could be taken over by the third instrument at the fourth arm, but this would impair the course of the operation because frequent changes of the two instruments controlled by the surgeon’s right hand would be necessary. In presence of a second console surgeon, the fourth arm could also be used for dynamic assistance – but this requires the availability of a second console. Since the arms must not be used outside the field of vision due to the lack of haptic feedback, the thread must be released and gripped more frequently. These problems are solved by using the self-fixing barbed suture material, which ideally complements robotic surgery. The suture is tightened once in a controlled manner and then maintains its tension. A continuously constant tension is thus guaranteed despite the lack of retraction. In addition, the final knot is not necessary, although technically this is easily possible with the help of the robot. We consider the 15-cm long thread to be appropriate. Longer threads make the guidance more difficult. Because of the better visibility, we use the colored version. It should be noted that after completion of the anastomosis, the thread should be cut as short as allowable to avoid local adhesions. Overlong threads carry the risk of developing an acute intestinal obstruction [22].

Another possible advantage of using the barbed suture is a gain in time; however, we cannot judge this due to the lack of a control group. Vidarsson et al. 2017 [7] and Pennestri et al. 2018 [8] describe a significant time gain using barbed sutures in laparoscopic RYGB procedures, whereas Gys et al. 2017 found significant time benefit only for the anastomosis itself, not for the total procedure time [6]. Compared to a historical laparoscopic RYGB cohort [12], the mean operative time with the surgical robot was about 10 min shorter. As yet, we are unable to provide a comparison with a robotic gastric bypass using Vicryl (Ethicon, Johnson & Johnson, Cincinnati, OH, USA). Compared to previous studies [16–20], which used vicryl in robotic RYGB, our average operative time of 121 min (robotic proximal RYGB, Additional file 1) is much shorter (Ahmad 2016 155 min, Benizri 2013 130 min, Buchs 2014 245 min, Hagen 2011 293 min / mean operative time, robotic RYGB). However, the anastomosis techniques are not comparable. Instead of a robotic-sewn gastrojejunostomy, we regularly performed a side-to-side linear anastomosis.

The use of self-fixing sutures may reduce the learning curve of bypass surgery [23]. Our own experience shows that in the course of the 37 proximal RYGB operations, a continuous improvement in the mean operation time was achieved. To what extent the use of the suture material had an influence on the learning curve cannot be assessed. In our opinion, this is rather the result of the increasing experience with the da Vinci Surgical System.

Our data show that the use of barbed sutures in robotic RYGB surgery is safe. No intraoperative complications occurred. No thread ruptures were observed. While the weight course was comparable to the expected weight loss, our cohort showed no insufficiency nor stenosis of the anastomosis. Due to omentum necrosis, one case was subjected to a relaparoscopy and partial omentum resection. This complication was not related to the selected suture material or the use of the da Vinci Surgical System.

It should be noted that the cost of a Stratafix thread is many times higher than the cost of a Vicryl thread (€23.04 against €1.80, Ethicon, Johnson & Johnson, Cincinnati, OH, US). At a cost of around €15 per minute for an operating theatre in our hospital, the investment would pay off if there was a time gain of 1.5 min. We assume that this is the case, even though the previous data do not yet allow this conclusion.

In our opinion, the use of self-fixing sutures is an ideal complement to the surgical robot. Even without permanent retraction via a third instrument using the fourth robotic arm or the assistant, the suture promises a continuously constant tension. Due to the above-mentioned advantages, the suture material is now being exclusively used in laparoscopic as well as robotic gastric bypass and is increasingly established in other areas as well in our clinic. According to the manufacturer Ethicon, Johnson & Johnson (Cincinnati, OH, USA), there is no contraindication regarding the use of the suture material in gastrointestinal anastomoses, but the manufacturer has not yet tested its safety and effectiveness so far. This makes corresponding studies all the more important, as the material is already widely used.

Although we are the first to report on the use of barbed sutures in robotic bariatric bypass surgery, it must be noted that our cohort is relatively small with a missing control group. All procedures were performed only by a single surgeon using a linear stapling technique. Whether the use of barbed sutures has advantages over conventional sutures cannot be answered by this study. Furthermore, the case series does not allow a statement on cost efficiency, time efficiency, or effects on the learning curve.

## Conclusions

The results of our study indicate that use of self-fixing barbed sutures in performing intestinal anastomosis during the robotic RYGB is safe and appropriate. Further studies with higher evidence levels are required.

## Additional files

Additional file 1: Peri- and postoperative results after using barbed sutures in robotic bariatric bypass surgery. Operative time, robotic time, length of hospital stay, and ideal body weight was calculated as that equivalent to a BMI of 25 kg/m^2^. Follow-up rate 98%, number of reoperations and complications within the first 30 days according to Clavien-Dindo classification [21]. (DOCX 16 kb)

Additional file 2: Complete dataset of robotic RYGB surgeries. (CSV 3 kb)

## Data Availability

The datasets used and analysed during the current study are available from the corresponding author on reasonable request. All operations performed have been registered in the national registry of the German Society for General and Visceral Surgery (StuDoQ|MBE).

## Abbreviations

BMI: body mass index
EOSS: Edmonton obesity staging system
RYGB: Roux-en-Y gastric bypass

## References

[1] Buchwald H, Avidor Y, Braunwald E, Jensen MD, Pories W, Fahrbach K (2004). Bariatric surgery: a systematic review and meta-analysis. JAMA..

[2] Angrisani L, Santonicola A, Iovino P, Vitiello A, Higa K, Himpens J (2018). IFSO worldwide survey 2016: primary, Endoluminal, and Revisional procedures. Obes Surg.

[3] Giordano S, Salminen P, Biancari F, Victorzon M (2011). Linear stapler technique may be safer than circular in gastrojejunal anastomosis for laparoscopic roux-en-Y gastric bypass: a meta-analysis of comparative studies. Obes Surg.

[4] Abellán I, López V, Lujan J, Abrisqueta J, Hernández Q, Frutos MD (2015). Stapling versus hand suture for Gastroenteric anastomosis in roux-en-Y gastric bypass: a randomized clinical trial. Obes Surg.

[5] Gys B, Gys T, Ruyssers M, Lafullarde T (2017). Laparoscopic linear stapled running Enterotomy closure in roux-en-Y gastric bypass using absorbable unidirectional barbed suture (Stratafix® 2/0). Obes Surg.

[6] Gys B, Gys T, Lafullarde T (2017). The use of unidirectional knotless barbed suture for Enterotomy closure in roux-en-Y gastric bypass: a randomized comparative study. Obes Surg.

[7] Vidarsson Bjarni, Sundbom Magnus, Edholm David (2017). Shorter overall operative time when barbed suture is used in primary laparoscopic gastric bypass: A cohort study of 25,006 cases. Surgery for Obesity and Related Diseases.

[8] Pennestrì Francesco, Gallucci Pierpaolo, Prioli Francesca, Giustacchini Piero, Ciccoritti Luigi, Sessa Luca, Bellantone Rocco, Raffaelli Marco (2018). Barbed vs conventional sutures in bariatric surgery: a propensity score analysis from a high-volume center. Updates in Surgery.

[9] Economopoulos Konstantinos P., Theocharidis Vasileios, McKenzie Travis J., Sergentanis Theodoros N., Psaltopoulou Theodora (2015). Robotic vs. Laparoscopic Roux-En-Y Gastric Bypass: a Systematic Review and Meta-Analysis. Obesity Surgery.

[10] Li Kun, Zou Jianan, Tang Jianxiong, Di Jianzhong, Han Xiaodong, Zhang Pin (2016). Robotic Versus Laparoscopic Bariatric Surgery: a Systematic Review and Meta-Analysis. Obesity Surgery.

[11] Sebastian Raul, Howell Melanie H., Chang Kai-Hua, Adrales Gina, Magnuson Thomas, Schweitzer Michael, Nguyen Hien (2018). Robot-assisted versus laparoscopic Roux-en-Y gastric bypass and sleeve gastrectomy: a propensity score-matched comparative analysis using the 2015–2016 MBSAQIP database. Surgical Endoscopy.

[12] Beckmann J. H., Aselmann H., Egberts J. H., Bernsmeier A., Laudes M., Becker T., Schafmayer C., Ahrens M. (2018). Roboterassistierter vs. laparoskopischer Magenbypass. Der Chirurg.

[13] Egberts J-H, Möller T, Hauser C, Beckmann J-H, Schlemminger M, Becker T (2018). Robotic assisted sleeve lobectomy with the use of barbed sutures. J Vis Surg [Internet].

[14] Aselmann H., Egberts J. Hendrik, Beckmann J. Henrik, Stein H., Schafmayer C., Hinz S., Reichert B., Becker T. (2017). Roboterassistierte pyloruserhaltende Pankreaskopfresektion. Der Chirurg.

[15] Li H, Liu C, Zhang H, Xu W, Liu J, Chen Y (2015). The use of unidirectional barbed suture for urethrovesical anastomosis during robot-assisted radical prostatectomy: a systematic review and meta-analysis of efficacy and safety. PLoS One.

[16] Ahmad Arif, Carleton Jared D., Ahmad Zoha F., Agarwala Ashish (2015). Laparoscopic versus robotic-assisted Roux-en-Y gastric bypass: a retrospective, single-center study of early perioperative outcomes at a community hospital. Surgical Endoscopy.

[17] Benizri Emmanuel I., Renaud Myriam, Reibel Nicolas, Germain Adeline, Ziegler Olivier, Zarnegar Rasa, Ayav Ahmet, Bresler Laurent, Brunaud Laurent (2013). Perioperative outcomes after totally robotic gastric bypass: a prospective nonrandomized controlled study. The American Journal of Surgery.

[18] Buchs Nicolas C., Morel Philippe, Azagury Dan E., Jung Minoa, Chassot Gilles, Huber Olivier, Hagen Monika E., Pugin François (2014). Laparoscopic Versus Robotic Roux-En-Y Gastric Bypass: Lessons and Long-Term Follow-Up Learned From a Large Prospective Monocentric Study. Obesity Surgery.

[19] Hagen Monika E., Pugin Francois, Chassot Gilles, Huber Olivier, Buchs Nicolas, Iranmanesh Pouya, Morel Philippe (2011). Reducing Cost of Surgery by Avoiding Complications: the Model of Robotic Roux-en-Y Gastric Bypass. Obesity Surgery.

[20] Scozzari Gitana, Rebecchi Fabrizio, Millo Paolo, Rocchietto Stefano, Allieta Rosaldo, Morino Mario (2010). Robot-assisted gastrojejunal anastomosis does not improve the results of the laparoscopic Roux-en-Y gastric bypass. Surgical Endoscopy.

[21] Dindo D, Demartines N, Clavien PA (2004). Classification of surgical complications: a new proposal with evaluation in a cohort of 6336 patients and results of a survey. Ann Surg.

[22] Jang Sung Ho, Jung Yun Kyung, Choi Sung Ji, Ha Tae Kyung (2017). Postoperative mechanical small bowel obstruction induced by V-Loc barbed absorbable suture after laparoscopic distal gastrectomy. Annals of Surgical Treatment and Research.

[23] Kassir R, Blanc P, Breton C, Tiffet O, Iannelli A, Ben AI (2014). Laparoscopic roux-en-Y gastric bypass with the absorbable bidirectional monofilament barbed suture Stratafix®: the hand-sewn technique. Obes Surg.

