# Supplementary material for: Use of barbed sutures in robotic bariatric bypass surgery: a single-center case series
Source: BMC Surg. 2019 Jul 23;19:97. doi: 10.1186/s12893-019-0563-z (PMC6651907; doi:10.1186/s12893-019-0563-z)
Supplement: Supplementary file 1 — Peri- and postoperative results after using barbed sutures in robotic bariatric bypass surgery. Operative time, robotic time, length of hospital stay, and ideal body weight was calculated as that equivalent to a BMI of 25 kg/m2. Follow-up rate 98%, number of reoperations and complications within the first 30 days according to Clavien-Dindo classification [21]. (DOCX 16 kb) [file 12893_2019_563_MOESM1_ESM.docx]

**Additional File 1**. Peri- and postoperative results after using barbed sutures in robotic bariatric bypass surgery. Operative time, robotic time, length of hospital stay, and ideal body weight was calculated as that equivalent to a BMI of 25 kg/m^2^. Follow-up rate 98%, number of reoperations and complications within the first 30 days according to Clavien-Dindo classification [21].

|  | n | Operative time/h | Robotic time/h | LOS/d | %TWL 30 days | %EWL 30 days | Follow up, n | Reoperations | Clavien Dindo 0 | Clavien Dindo 1 | Clavien Dindo 2 | Clavien Dindo 3a | Clavien Dindo 3b | Clavien Dindo 4a -5 |
| --- | --- | --- | --- | --- | --- | --- | --- | --- | --- | --- | --- | --- | --- | --- |
| Robotic prox. RYGB | 37 | 02:00:47±00:30:06 | 01:31:29±00:23:14 | 4.1±1.0 | 9.9±2.9 | 21.9±7.5 | 37 (100%) | 1 (3%) | 34 (92%) | 1 (3%) | 0 | 1 (3%) | 1 (3%) | 0 |
| Robotic prox. RYGB ReDo | 4 | 03:10:15±01:05:27 | 02:40:45±01:09:08 | 5.0±1.4 | 5.5±3.2 | 19.3±10.2 | 4 (100%) | 0 | 4 (100%) | 0 | 0 | 0 | 0 | 0 |
| Robotic dist. RYGB ReDo | 9 | 02:06:20±00:22:17 | 01:37:00±00:16:31 | 4.3±0.7 | 6.3±3.6 | 14.1±6.9 | 8 (89%) | 0 | 7 (88%) | 0 | 1 (13%) | 0 | 0 | 0 |
| All | 50 | 02:07:20±00:36:57 | 01:38:01±00:32:59 | 4.2±1.0 | 9.0±3.4 | 20.4±8.0 | 49 (98%) | 1 (2%) | 45 (92%) | 1 (2%) | 1 (2%) | 1 (2%) | 1 (2%) | 0 |

Values are expressed as mean ± standard deviation. Percentages have been added to categorical values. Robotic prox. RYGB: DaVinci proximal Roux-en-Y gastric bypass; Robotic prox. RYGB ReDo: DaVinci proximal Roux-en-Y gastric bypass as secondary procedure; Robotic dist. RYGB ReDo: DaVinci distal Roux-en-Y gastric bypass as secondary procedure. LOS, length of hospital stay. %TWL, percentage total weight loss. %EWL, percentage excess weight loss.
